# Prevalence and Associated Factors of Physical Complaints Among Japanese Esports Players: A Cross-Sectional Study

**DOI:** 10.7759/cureus.66496

**Published:** 2024-08-09

**Authors:** Takafumi Monma, Takashi Matsui, Shoya Koyama, Hiromasa Ueno, Junichi Kagesawa, Chisato Oba, Kentaro Nakamura, Hideki Takagi, Fumi Takeda

**Affiliations:** 1 Institute of Health and Sport Sciences, University of Tsukuba, Ibaraki, JPN; 2 Research and Development Center for Sport Innovation, University of Tsukuba, Ibaraki, JPN; 3 Research and Development Center for Lifestyle Innovation, University of Tsukuba, Ibaraki, JPN; 4 Esports Business, REJECT Inc., Tokyo, JPN; 5 Esports Business, NTTe-Sports Inc., Tokyo, JPN; 6 Food Microbiology and Function Research Laboratories, Research and Development Division, Meiji Co. Ltd., Tokyo, JPN

**Keywords:** public health, musculoskeletal disease, somatic symptom, video game, electronic sports

## Abstract

Background

In the evolving landscape of electronic sports (esports), where economic and social expectations are soaring, a critical concern has emerged in physical complaints among esports players. However, empirical insights into these complaints’ prevalence and influencing factors are scarce. This study aimed to determine the prevalence of physical complaints and their association with esports activities among Japanese esports players.

Methodology

A web-based, cross-sectional survey encompassing 175 esports players from both professional and amateur teams in Japan was conducted. The analysis focused on 79 male participants (average age: 21.6 ± 5.6 years) with complete responses. The survey items included the esports factors about the esports title mainly play (device, career duration, playing time per day on weekdays and holidays, and the distance between the screen and the face) and physical complaints (headache, neck pain, stiff or sore shoulders, wrist pain, finger pain, lower back pain, and eye fatigue).

Results

A total of 49.4% reported stiff or sore shoulders, 48.1% faced eye fatigue, and 30.4% had headaches. Professionals exhibited a significantly higher likelihood of neck, wrist, and lower back pain and eye fatigue than amateurs. Age-adjusted univariate logistic regression analysis uncovered that using mainly mobile devices and being closer to the screen and face were significantly associated with neck pain, stiff or sore shoulders, lower back pain, and eye fatigue.

Conclusions

These results suggest that poor posture caused by using mobile devices and being closer to the screen was related to various physical complaints.

## Introduction

Electronic sports (esports) is “an organized and competitive approach to playing computer games” [1] through various platforms (such as consoles, computers, mobile devices, and virtual reality). The global esports audience and the number of active esports players increase annually [2]. Large-scale competitions are increasing, and the International Olympic Committee officially launched its Olympic Esports Series in 2023 [3]. In Japan, esports events have been the programs of the National Sports Festival since 2019 [4], and expectations of the economic benefits and social significance of esports are growing [5].

The growing interest in esports has resulted in some considerable downsides. Physical health is a major concern. Excessive video game playing is associated with physical complaints [6,7]. Additionally, previous studies that investigated the prevalence of physical complaints among competitive esports players have reported that approximately half of esports players have eye fatigue [8], headache [9], neck pain [10,11], lower back pain [11], and hand/wrist pain [11].

In general, the proportion of people with physical complaints varies by attribute, such as age and body size [12,13]. Moreover, similar to traditional sports [14], there may be differences in musculoskeletal conditions between professionals, who have periodized training schedules, access to team resources, and season-based play, and amateurs [15]. However, differences in the proportion of those with physical complaints by attribute, such as age, body size, and esports level (professional or amateur), have not been examined among esports players.

The issue of physical complaints in esports players is the same as in the context of occupational health; esports practice requires long periods of computer and console use while sitting in poor postures, repetitive or forceful movements, and prolonged screen exposure [16,17]. However, few previous studies have examined the relationship between esports activities and physical complaints, and their findings are not consistent. Cross-sectional studies in professional esports players [9] and collegiate esports players [18] reported that those who spent more time practicing had a higher incidence of injury, while another study showed that esports players with musculoskeletal pain participated in less esports training than those without pain [19]. A study in Saudi Arabia showed that the duration of playing video games was inversely associated with musculoskeletal disorders, with gamers who played for 1-5 years showing a higher rate of musculoskeletal disorders than those who played for more than 10 years [11]. However, another study reported that career duration was not significantly associated with ease of eyestrain or leg numbness [10]. In addition, other factors of esports activities, such as the device used for esports and posture during play, may be related to physical complaints in light of the points made above, but no empirical studies have been conducted.

Therefore, this study aimed to determine the prevalence of physical complaints and their association with esports activities among Japanese esports players.

This article was previously posted to the medRxiv preprint server on November 14, 2023 [20].

## Materials and methods

Procedure and participants

In this cross-sectional study, research cooperation was obtained from one professional team and five amateur teams in Japan. This professional team was incorporated and operated by generating revenue through sponsorships, player video streaming, media appearances, and merchandising. The players of this professional team participated in professional leagues and tournaments. In contrast, the amateur teams were formed by volunteers, were not incorporated, and could not operate on a large scale similar to the professional team. Representatives of each team were asked to survey all their team members. In total, 175 esports players were surveyed. An anonymous, self-administered, web-based questionnaire survey was conducted from April to May 2021. In total, 100 responses were collected (response rate: 57.1%). Of these, no women were on the professional team, and only 19 were on the amateur teams. Therefore, women were excluded from the analysis. Furthermore, two men were excluded from the analysis owing to incomplete responses. Finally, 79 men with complete responses were included in the analysis.

This study was approved by the Ethics Committee of the Institute of Health and Sport Sciences, University of Tsukuba, in compliance with the Declaration of Helsinki (reference number: Tai 021-112). All respondents provided informed consent to participate in the study. On the first page of the survey form, we outlined the significance and purpose of the survey, stated that participation is voluntary and that there would be no disadvantage for choosing not to participate, emphasized that there are no right or wrong answers, and assured that personal information will be protected. Respondents read these ethical considerations and proceeded with the survey only if they agreed to them.

Questionnaires

The questionnaire included questions on attributes, esports activities, and physical complaints. The attributes included age, height, weight, and team affiliation. Body mass index (BMI) was calculated from height and weight. BMI was classified into the following three groups based on the World Health Organization’s definitions [21]: under 18.5 kg/m^2^ was considered “underweight,” 18.5 kg/m^2^ to under 25 kg/m^2^ as “normal weight,” and 25 kg/m^2^ or above as “overweight or obese.” Furthermore, the esports level was categorized as professional or amateur based on the teams to which they belonged.

Regarding esports activities, questions were asked about the esports titles the respondents were primarily playing, including the primary esports title, career duration, playing time per day on weekdays and holidays, and the distance between the screen and the face. Based on the response of the primary esports title, the device was categorized as either “PC or console” or “mobile.”

Regarding physical complaints, the following seven items were established that had been prevalent among esports players in previous studies [8-11,18,19]: headache, neck pain, stiff or sore shoulders, wrist pain, finger pain, lower back pain, and eye fatigue. Respondents were asked about the presence or absence of each item.

Statistical analysis

The esports activities and presence of each physical complaint were calculated and compared by age (divided by the median), BMI category, and esports level (professional or amateur) using chi-square tests, Fisher’s exact tests, Mann-Whitney U tests, and Kruskal-Wallis tests. After confirming associations between each factor of esports activities using Mann-Whitney U tests and Spearman’s rank correlation analyses, age-adjusted univariate logistic regression analyses were conducted with each physical complaint as the dependent variable and esports activities as the independent variables. This analysis used physical complaints reported by 10% or more of the respondents. The distance between the face and the screen was converted to 10 cm to avoid an extremely small range of odds ratios (ORs). Data analysis was performed using SPSS Statistics, version 29.0 (IBM Corp., Armonk, NY, USA), and the statistical significance level was set at 5%.

## Results

Table 1 presents the characteristics of the respondents. The mean age was 21.6 ± 5.6 years. Of the respondents, 39 (49.4%) were professionals and 40 (50.6%) were amateurs. Regarding the device, 60 (75.9%) respondents mainly used PCs or consoles, and 19 (24.1%) used mobiles.

**Table 1:** Respondent characteristics.

		Mean ± SD or n (%)
Attributes		
Age		21.6 ± 5.6
Body mass index	Underweight	27 (34.2)
	Normal weight	43 (54.4)
	Overweight or obese	9 (11.4)
Esports level	Professional	39 (49.4)
	Amateur	40 (50.6)
Esports activities		
Esports title	VALORANT	5 (6.3)
	Rainbow Six Siege	7 (8.9)
	PUBG	5 (6.3)
	APEX Legends	8 (10.1)
	Call of Duty	3 (3.8)
	Call of Duty mobile	5 (6.3)
	PUBG Mobile	7 (8.9)
	PUBG Mobile (Academy)	3 (3.8)
	League of Legends Wild Rift	4 (5.1)
	Fortnite	32 (40.5)
Career duration (years)		2.4 ± 1.5
Playing time per day (hours)	Weekdays	4.2 ± 2.9
	Holidays	4.1 ± 3.3
Distance between the screen and the face (cm)		44.7 ± 26.7
Physical complaints (presence)		
Headache		24 (30.4)
Neck pain		23 (29.1)
Stiff or sore shoulders		39 (49.4)
Wrist pain		5 (6.3)
Finger pain		3 (3.8)
Lower back pain		23 (29.1)
Eye fatigue		38 (48.1)

Among physical complaints, stiff or sore shoulders were the most common, with 39 (49.4%) respondents, followed by eye fatigue with 38 (48.1%) respondents, headache with 24 (30.4%) respondents, and neck pain and lower back pain with 23 (29.1%) respondents each.

Table 2 shows the esports activities and proportion of each physical complaint by age, BMI category, and esports level (professional or amateur). Professionals were more likely to use mainly mobile devices than amateurs (p < 0.001). Furthermore, professionals spent significantly more time on esports play on weekdays (p < 0.001) and had a significantly shorter distance between the face and screen (p < 0.001) than amateurs. Regarding physical complaints, professionals were significantly more likely to experience neck pain (p < 0.05), wrist pain (p < 0.05), lower back pain (p < 0.05), and eye fatigue (p < 0.01) than amateurs. There was no difference in esports activities or physical complaints according to age or BMI category.

**Table 2:** Esports activities and physical complaints by attributes. IQR: interquartile range ^a^: Chi-square test; ^b^: Fisher’s exact test; ^c^: Mann–Whitney U test; ^d^: Kruskal–Wallis test

		Age			Body mass index				Esports level		
		<20 years	≥20 years	P-value	Underweight	Normal weight	Overweight or obese	P-value	Professional	Amateur	P-value
		n (%) or median [IQR]	n (%) or median [IQR]		n (%) or median [IQR]	n (%) or median [IQR]	n (%) or median [IQR]		n (%) or median [IQR]	n (%) or median [IQR]	
Esports activities											
Device	PC or console	26 (70.3)	34 (81.0)	0.268^a^	18 (66.7)	34 (79.1)	8 (88.9)	0.312^a^	20 (51.3)	40 (100.0)	<0.001^b^
	Mobile	11 (29.7)	8 (19.0)		9 (33.3)	9 (20.9)	1 (11.1)		19 (48.7)	0 (0.0)	
Career duration (years)		2.5 [1.6]	2.4 [2.3]	0.425^c^	2.5 [2.3]	2.5 [1.7]	1.9 [3.3]	0.447^d^	2.8 [2.4]	2.4 [1.2]	0.906^c^
Playing time per day (hours)	Weekdays	4.0 [3.5]	4.0 [4.0]	0.162^c^	4.0 [3.0]	3.1 [6.0]	4.0 [5.5]	0.693^d^	6.0 [4.0]	2.8 [4.0]	<0.001^c^
	Holidays	4.0 [6.3]	4.0 [5.3]	0.835^c^	4.0 [6.0]	3.0 [6.5]	4.0 [5.5]	0.433^d^	4.0 [5.0]	3.5 [7.0]	0.334^c^
Distance between the screen and the face (cm)		40.0 [40.0]	40.0 [35.0]	0.906^c^	25.0 [30.0]	50.0 [30.0]	40.0 [30.0]	0.093^d^	25.0 [23.0]	52.5 [35.0]	<0.001^c^
Physical complaints											
Headache	Presence	10 (27.0)	14 (33.3)	0.543^a^	7 (25.9)	15 (34.9)	2 (22.2)	0.622^a^	12 (30.8)	12 (30.0)	0.941^a^
Neck pain	Presence	8 (21.6)	15 (35.7)	0.169^a^	10 (37.0)	11 (25.6)	2 (22.2)	0.525^a^	16 (41.0)	7 (17.5)	0.021^a^
Stiff or sore shoulders	Presence	15 (40.5)	24 (57.1)	0.141^a^	13 (48.1)	23 (53.5)	3 (33.3)	0.621^b^	23 (59.0)	16 (40.0)	0.092^a^
Wrist pain	Presence	2 (5.4)	3 (7.1)	1.000^b^	3 (11.1)	2 (4.7)	0 (0.0)	0.355^b^	5 (12.8)	0 (0.0)	0.026^b^
Finger pain	Presence	1 (2.7)	2 (4.8)	1.000^b^	2 (7.4)	1 (2.3)	0 (0.0)	0.692^b^	2 (5.1)	1 (2.5)	0.615^b^
Low back pain	Presence	14 (37.8)	9 (21.4)	0.109^a^	10 (37.0)	12 (27.9)	1 (11.1)	0.322^a^	16 (41.0)	7 (17.5)	0.021^a^
Eye fatigue	Presence	15 (40.5)	23 (54.8)	0.207^a^	14 (51.9)	20 (46.5)	4 (44.4)	0.949^b^	25 (64.1)	13 (32.5)	0.005^a^

Table 3 presents the relationships between each factor of esports activities. The device had a significant relation to the playing per day on weekdays (U = 366.5, Z = 2.350, p < 0.05) and the distance between the screen and the face (U = 111.5, Z = 5.278, p < 0.001). Playing time per day on weekdays also had a significant correlation with that of esports activities per day on holidays (ρ = 0.524, p < 0.001) and the distance between the screen and the face (ρ = -0.499, p < 0.001).

**Table 3:** Relationships between esports activities. *: p < 0.05; **: p < 0.001

	Device		Career duration	Playing time per day on weekdays	Playing time per day on holidays
	U	Z	ρ	ρ	ρ
Device					
Career duration	410.0	1.838			
Playing time per day on weekdays	366.5	2.350*	0.011		
Playing time per day on holidays	494.0	0.880	-0.068	0.524**	
Distance between the screen and the face	111.5	5.278**	0.073	-0.499**	-0.16

Table 4 shows the results of age-adjusted univariate logistic regression analyses, with each physical complaint as the dependent variable and esports activities as the independent variables. The device was significantly related to neck pain (OR = 5.62, 95% confidence interval (CI) = 1.72-18.33, p < 0.01), stiff or sore shoulders (OR = 7.63, 95% CI = 2.16-27.03, p < 0.01), lower back pain (OR = 4.71, 95% CI = 1.52-14.61, p < 0.01), and eye fatigue (OR = 4.45, 95% CI = 1.37-14.41, p < 0.05). The distance between the face and the screen also had a significant association with neck pain (OR = 0.61, 95% CI = 0.44-0.85, p < 0.01), stiff or sore shoulders (OR = 0.69, 95% CI = 0.54-0.87, p < 0.01), lower back pain (OR = 0.75, 95% CI = 0.59-0.96, p < 0.05), and eye fatigue (OR = 0.71, 95% CI = 0.56-0.89, p < 0.01).

**Table 4:** Relationships between esports activities and physical complaints. Age-adjusted logistic regression analysis. OR: odds ratio; CI: confidence interval

		Headache			Neck pain			Stiff or sore shoulders			Lower back pain			Eye fatigue		
		OR	95% CI	P-value	OR	95% CI	P-value	OR	95% CI	P-value	OR	95% CI	P-value	OR	95% CI	P-value
Device (0 = PC or console; 1 = mobile)		0.81	0.25-2.65	0.723	5.62	1.72-18.33	0.004	7.63	2.16-27.03	0.002	4.71	1.52-14.61	0.007	4.45	1.37-14.41	0.013
Career duration		1.07	0.79-1.46	0.648	1.17	0.85-1.60	0.337	1.03	0.77-1.37	0.865	1.12	0.81-1.54	0.497	0.83	0.61-1.12	0.219
Playing time per day	Weekdays	0.98	0.83-1.16	0.813	1.09	0.91-1.29	0.343	0.98	0.84-1.15	0.804	1.20	1.00-1.44	0.056	1.02	0.87-1.20	0.777
	Holidays	0.96	0.83-1.12	0.631	0.94	0.81-1.10	0.464	0.94	0.82-1.08	0.410	0.93	0.79-1.08	0.339	0.92	0.80-1.06	0.245
Distance between the face and screen		0.91	0.75-1.12	0.372	0.61	0.44-0.85	0.003	0.69	0.54-0.87	0.002	0.75	0.59-0.96	0.024	0.71	0.56-0.89	0.003

## Discussion

This study aimed to determine the prevalence of physical complaints and their association with esports activities among Japanese esports players. The results showed that stiff or sore shoulders were the most common, followed by eye fatigue, headache, neck pain, and lower back pain. These proportions were similar to previous studies [8,10]. In contrast, wrist and finger pain was reported as relatively less frequent. Previous studies have shown a wide range of wrist, hand, or finger pain prevalence, ranging from 5% to 44.8% [8-11,18,19]. Unlike other body parts, the wrists and fingers require repetitive movements during esports activities. These repetitive movements may underpin wrist and finger pain. However, our study and these previous studies did not investigate repetitive movements of the wrist and finger; thus, future studies should consider them.

The proportion of those with neck, wrist, and lower back pain and eye fatigue was significantly higher among professionals than amateurs. In traditional sports, professionals with periodized training schedules and access to team resources are less prone to musculoskeletal problems than amateurs [14]. Our opposite result in esports players suggests an inadequate support system for professionals.

Using mobile devices and being closer to the screen were associated with neck pain, stiff or sore shoulders, lower back pain, and eye fatigue in the age-adjusted logistic regression analysis. Some systematic reviews have suggested associations between the regular use of mobile devices and physical complaints in the eyes, neck, and upper extremities [22-24]. Furthermore, these two would be linked problems because there was a relatively strong relationship between using mobile devices and being closer to the screen.

One of the reasons that these two esports activity factors were associated with physical complaints could be due to the player’s posture when playing esports. Mobile esports players are more likely to adopt a forward or flexed head posture for better eye focus, while the elbows press on the table to hold the arm and the mobile device. They may also have a slouched posture and rounded shoulders. Such poor posture can impose problems on the musculoskeletal and ocular systems [25,26].

Based on our results, ergonomic approaches can effectively prevent physical complaints. Height-adjustable desks or desk-mounted armrests can help position the arm or mobile device higher or closer to the upper extremities. Increasing the strength and flexibility of the trunk muscles may also help address physical complaints. A previous study evaluated spinal function using an external noninvasive device and reported that professional mobile esports players have significantly poorer spine posture, mobility, and stability than the standard reference [27]. Moreover, offering opportunities to stand during esports activities may efficiently alleviate physical complaints. Although standing up in the middle of a set of games is difficult, providing opportunities to stand between sets is possible. Periodic interruption of sedentary behavior improves musculoskeletal symptoms [28]. Taking a break while using electronic equipment also reduces computer vision syndromes, including eye fatigue [29]. As esports players require long hours of esports play to enhance their competitive performance, these practical countermeasures for physical complaints, other than playtime limitations, are useful.

Career duration and playing time per day on weekdays and holidays had no significant association with physical complaints. A previous study reported that career duration was not significantly associated with ease of eyestrain or leg numbness [10], consistent with the findings of this study. However, another study in Saudi Arabia showed that the duration of playing video games was inversely related to musculoskeletal disorders, with gamers who played for 1-5 years showing a higher rate of having musculoskeletal disorders than those who played for more than 10 years [11]. Moreover, some previous studies reported those who spent more time practicing had a higher incidence of injury [9,18], while another study showed that esports players with musculoskeletal pain participated in less esports training than those without pain [19]. The discrepancy in these findings is likely due to the coexistence of two reasons, i.e., that the longer the career duration and playtime, the more likely it is for esports players to have physical complaints, and that esports players who have physical complaints cannot continue playing esports for longer durations or extended periods. Therefore, a longitudinal study is necessary to verify the causal relationships among these variables.

This study had some limitations. First, as noted above, as the number of respondents in this study was small, further studies are needed to target more esports players. Second, this study only involved one professional team and five amateur teams, and the response rate was relatively low. Therefore, the possibility of sampling bias cannot be ruled out. Third, this study used cross-sectional data; therefore, we could not evaluate the causal relationship between esports activities and physical complaints. A longitudinal study is necessary to verify the causal relationships among these variables. Fourth, other factors related to esports activities should also be considered. For example, equipment characteristics and repetitive movements of the wrists and fingers may be related to physical complaints. Fifth, our study only included male players. Currently, there are a few female esports players [30]. However, the number of women may increase in the future because physical attributes are unrelated to high performance in esports, allowing men and women to compete in the same events, unlike traditional sports [31]. Therefore, it is necessary to accumulate knowledge about female esports players. Finally, data were collected using self-reported questionnaires. Therefore, reporter bias cannot be ruled out.

Despite these limitations, to our knowledge, this is the first study to examine factors of esports activities from multiple perspectives related to physical complaints among esports players. As esports players require long hours of esports activities to enhance their competitive performance, it is notable that we were able to identify clues to practical countermeasures for their physical complaints from factors other than the duration of esports activities.

## Conclusions

Japanese esports players reported that stiff or sore shoulders were the most common complaint, followed by eye fatigue, headache, neck pain, and lower back pain. Mainly using mobile devices and being closer to the screen and face were associated with a higher proportion of those with neck pain, stiff or sore shoulders, low back pain, and eye fatigue. These results suggest that posture during esports activities is more strongly related to physical complaints than extended playing time. Therefore, ergonomic approaches might be an effective countermeasure.
